# Weight status at age 18 influences marriage prospects. A population-based study of Swedish men

**DOI:** 10.1186/1471-2458-12-833

**Published:** 2012-09-28

**Authors:** Malin Kark, Nina Karnehed

**Affiliations:** 1 Child and Adolescent Public Health Epidemiology Group, Department of Public Health Sciences, Karolinska Institutet, Norrbacka, Stockholm, 171 76, Sweden

**Keywords:** Longitudinal study, Marital status, Men, Obesity status

## Abstract

**Background:**

In a longitudinal population-based study of the relationship between body mass index (BMI) in early adulthood and marital status at 40 years of age, obese men were half as likely to be married compared with men of normal weight. Significant associations between obesity and marital status among men in a longitudinal setting are novel findings.

**Methods:**

The study cohort comprised Swedish men born from 1951 to 1961. Height and weight at age 18 was gathered from the Military Service Conscription Register and information on marital status at 40 years of age was obtained from population registers by record-linkage using the unique personal identification number. The odds ratio (OR) for being married was calculated by polytomous logistic regression analysis adjusting for birth year, intellectual performance, education, country of birth, residential area, socioeconomic position in childhood and adulthood, parental education and muscle strength.

**Results:**

Our study included 486 599 Swedish men. Young men who were obese (BMI≥30.0) at 18 years of age had an OR of 0.49 (95% CI: 0.46–0.52) for being married at 40 years of age compared to normal weight men (BMI: 18.5–24.9). Underweight men (BMI≤18.5) had an OR of 0.84 (0.82–0.86) and overweight men (BMI: 25.0–29.9) had an OR of 0.83 (0.80–0.85) for being married at 40 years of age.

**Conclusion:**

Underweight, overweight and obese men were less likely to be married than their normal weight counterparts. Obese men had the lowest likelihood of being married. Stigmatization and discrimination may partly explain these findings, but further research is needed before firm conclusions can be drawn.

## Background

The prevalence of obesity has increased worldwide during the last few decades [1,2]. A growing body of literature indicates that obese people are stigmatized and discriminated against in a number of areas, including the workplace, school, social settings, and health care [3]. Marriage can be seen as a proxy of social success and previous longitudinal studies have reported a marriage market penalty for heavier individuals, showing that obese women, to a higher extent, than obese men are less likely to get married [4,5]. These studies have only been able to follow their study participants 10–15 years with regard to early weight status and marital status among men.

Married people seem to have lower morbidity and mortality compared to their non-married counterparts, while divorced people have the highest morbidity and mortality [6,7]. It has been suggested that selection mechanisms as well as protection mechanisms may explain some of the advantages of marriage [8,9]. Marriage may protect against poor health and mortality risks and healthier people might marry healthier counterparts [8]. Additionally, thinner women are more likely to be involved in romantic relationships [5]. However, a large body of research has found that women as well as men are heavier when married than prior to marriage, and also that married men are heavier than their single never married counterparts [5].

In light of these results we conducted a longitudinal population-based study of the relationship between body mass index (BMI) at age 18 and marital status at 40 years of age, at which age marital status is established, controlling for several important confounding factors such as socioeconomic position in childhood and adulthood. The study was conducted within a cohort in which socioeconomic disadvantage due to obesity among men has previously been reported [10,11].

## Methods

### Population and record linkage

The study cohort consisted of Swedish men born from 1951 to 1961 (n=528 770) who were identified in Statistic Sweden’s Register of the Total Population and were eligible for a military conscription examination at 18 years of age. During the period covered by this study, military conscription was compulsory for all men with Swedish citizenship, and over 90% of the male population underwent military conscription medical examinations. Men who did not participate in a military conscription examination for unknown reasons were excluded (7.5%). The additional exclusion of men who had been married before military conscription examination (0.5%) gave a final study population of 486 599 men. Those married prior to the examination did not differ from the men included in the study with regard to mean BMI (p-value>0.0001).

Information on the study subjects was obtained by record linkage between the Swedish Military Service Conscription Register, the Population and Housing Censuses (PHC) of 1960, 1970, 1975, 1980 and 1985 and the Longitudinal Database of Education, Income, and Occupation (LOUISE) of 1990–2004. Mortality data were obtained from the Cause of Death Register, and migration data were obtained from the Register of the Total Population. Parents of study cohort members were identified in the Multi-Generation Register using the unique personal identification number ascribed to all individuals with permanent residence in Sweden. We followed the index cohort and their parents through the registers mentioned above from birth until the end of 2004. The youngest cohort, born in 1961, was followed from birth to age 43 years and the oldest cohort, born in 1951, from birth to age 53 years.

### Outcome variables

Marital status refers to whether the subject was never married, married (not including a common law spouse), divorced or a widower, and was taken from LOUISE between the years 1990 to 2004 and from the PHC:s 1975, 1980 and 1985. Marital status are reported at 25, 30, 35, 40 and 45 years due to available information from PHC:s for the earlier ages. Information on marital status was available, yearly, only from 1990, when the oldest birth cohort born in 1951 was 39 years of age, we chose to present information on marital status at 40 years of age.

### Explanatory variables and potential confounders

Height and weight were measured at the military conscription examination. BMI was calculated as weight (kilograms) divided by squared height (metres squared) and categorized as underweight (BMI<18.5), normal weight (18.5≤BMI<25.0), overweight (25.0≤BMI<30.0), and obese (BMI≥30) according to the World Health Organization criteria [12]. There were six military conscription centres in Sweden, and the analyses were adjusted for these areas. Adjustments were also made for data on muscular strength, based on a weighted mean of hand grip, arm flexion and leg extension (Newton) measured during the military conscription examination. This was done in order to at least partly account for the inherent limitation of BMI as a measure of adiposity; namely its inability to distinguish fat-free mass from fat mass. Intellectual performance was measured using four subtests representing logical, spatial, verbal and technical abilities [13,14]. A global intelligence score was derived from a summation of the four subtests and was standardized, by the National Service Administration, to give a Gaussian distributed score between 1 and 9 [13,14]. Higher values indicate greater intellectual performance.

Data from PHC and LOUISE included level of education and socioeconomic position (both subject and parents) as well as the subjects’ own country of birth. Information on parental socioeconomic position was used as a proxy for childhood socioeconomic position. We used the highest socioeconomic position of the parent, either in PHC 1960 or PHC 1970, and whichever was nearest to age 10 years of the study subjects. Further, we used the highest level of education of the parent, in PHC 1970. Own level of socioeconomic position, from PHC, was taken at 30 years of age and own level of education was the highest education registered in the LOUISE. Socioeconomic groups were white-collar, blue-collar, self-employed including farmers, and other. Level of education was divided into low (9 years or less), medium (secondary school) and high (high school and higher). Ethnicity was categorized as being born in Sweden or in a foreign country. Residential area at the time of military conscription examination was categorized as urban, semi-urban and rural, as described in greater detail elsewhere [15].

Information on pre-existing psychiatric disorders was retrieved from the Hospital Discharge Register before the military conscription examination (data are available in partial form between 1968 and 1972 and in complete form from 1973) and information at the military conscription examination was extracted from the Swedish Military Service Conscription Register. Pre-existing psychiatric disorders were defined based on diagnoses according to ICD 8 (codes 290–319), ICD9 (codes 290–319) or ICD10 (codes F00–F99) both before and at the military conscription examination. The psychiatric disorders included different psychoses such as depression and schizophrenia, different neurotic disorders, personality disorders, mental disorders diagnosed in childhood and mental retardation. A dichotomized variable was created indicating a pre-existing psychiatric disorder or no such disorder.

### Statistical methods

Associations between BMI categories and marital status were estimated by polytomous logistic regression analyses using PROC TPHREG in SAS [16]. The polytomous logistic regression is similar to an ordinary logistic regression but uses a combined adjustment for confounding variables. The polytomous regression accommodates an outcome variable with more than two categories and compares each against a base. The base used in our analyses was the category “Never married”. The polytomous logistic regression models were adjusted for birth year, the military conscription centre, residential area, country of birth, education, socioeconomic position, intellectual performance, parental education, childhood socioeconomic position, muscle strength and psychiatric disease before or at the military conscription examination. We conducted a step-wise polytomous regression analysis with the above-mentioned variables (data not shown). The final model is shown under Results. The study was approved by the Regional Ethical Review Board at the Karolinska Institutet, Stockholm, Sweden.

## Results

**Figure 1:**
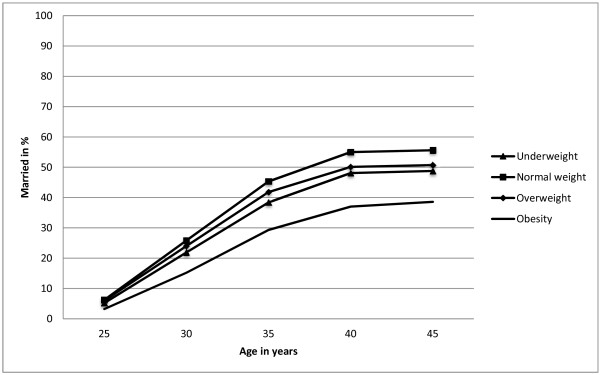
**Percentages of 25- to 45-year-old married men, born between 1951 and 1961 in Sweden, according to weight status at 18 years of age.** Marital status is taken between 1976 and 2006.

Figure 1 shows the proportion of men in the study cohort who married between the ages of 25 to 45 years of age, stratified according to weight status. Figure 1 shows that marriage was consistently less common among obese men than among normal weight men.

Table 1 shows the basic characteristics of the study population with regard to marital status at 40 years of age. Married men were more likely to have completed higher education (for the index subjects and their parents), to be white-collar workers (for the index subjects and their parents) and to have higher scores on the intellectual performance test. No substantial differences with regard to residential area or country of birth were found concerning marital status, whereas pre-existing psychiatric disorders were more common among men who had never married.

**Table 1:** **BMI, socio-demographic characteristics andpercentage according to maritalstatus at age 40(between 1991 and 2001)among men born between1951 and 1961 inSweden**

	**Number**	**Married**^**1**^** (%)**	**Never married**^**1**^** (%)**	**Divorced**^**1**^** (%)**	**Widowed**^**1**^** (%)**
**BMI category**^**2**^** (n=472 190)**					
Underweight	51 808	48.1	45.1	6.7	0.1
Normal weight	382 062	55.0	38.2	6.7	0.1
Overweight	33 101	50.1	43.0	6.8	0.1
Obese	5 219	37.0	57.0	5.9	0.1
**Education**^**1**^** (n=471 559)**					
High education	141 544	61.7	33.3	4.9	0.1
Medium education	231 605	51.2	41.3	7.4	0.1
Low education	98 410	48.4	43.8	7.7	0.1
**Socioeconomic position**^**3**^** (n=454 662)**					
White-collar	145 847	63.6	30.7	5.6	0.1
Farmers	7 517	58.5	38.2	3.1	0.2
Blue-collar	241 889	50.9	41.7	7.3	0.1
Other	59 409	40.9	50.9	8.1	0.1
**Intellectual performance**^**2**^** (n=471 714)**					
Lower	170 993	46.2	46.0	7.7	0.1
Medium	176 050	55.5	37.7	6.7	0.1
Higher	124 671	61.5	32.9	5.5	0.1
**Residential area**^**2**^** (n=470 047)**					
Urban	241 764	53.8	38.8	7.3	0.1
Semi-urban	164 805	54.2	39.4	6.3	0.1
Rural	63 478	51.7	42.5	5.7	0.1
**Country of birth**^**3**^** (n=472 093)**					
Sweden	463 422	53.8	39.4	6.7	0.1
Other	8 671	49.6	41.2	9.1	0.1
**Childhood socioeconomic position**^**3**^** (n=441 704)**					
White-collar	151 025	55.5	37.8	6.6	0.1
Farmers	36 333	57.3	38.4	4.1	0.2
Blue-collar	236 241	51.8	41.0	7.1	0.1
Other	18 105	46.4	45.0	8.5	0.1
**Parental education**^**3**^** (n=460 684)**					
High education	49 423	59.1	35.2	5.6	0.1
Medium education	138 772	54.3	38.6	7.0	0.1
Low education	272 489	52.5	40.6	6.8	0.1
**Psychiatric disorder**^**2,4**^** (n=472 190)**					
Yes	46 635	41.2	49.8	8.9	0.1
No	425 555	55.1	38.3	6.5	0.1
**Muscle strength**^**2**^** (n=472 051)**					
Low quintile	90 470	47.9	45.6	6.4	0.1
Low to mediate quintile	119 795	53.4	40.0	6.5	0.1
Mediate quintile	97 906	55.2	37.9	6.8	0.1
Mediate to high quintile	89 720	56.1	36.8	7.0	0.1
High quintile	74 160	56.5	36.4	7.0	0.1

n=number, ^1^=LOUISE, ^2^=Military Service Conscription Register, ^3^=Population and Housing Censuses, ^4^=Hospital Discharge Register.

Stepwise adjustment for possible confounders was performed (data not shown) and the final model included education, socioeconomic position, intellectual performance, country of birth, socioeconomic position during childhood, parental education, residential area, military conscription centre, presence of psychiatric disorder before and/or at military examination and muscle strength. Table 2 shows the OR for marital status at 40 years of age, taking confounding factors into account. Crude ORs (data not shown) didn’t differ to any larger extent from the adjusted ORs in Table 2. Obese men were significantly less likely to be married compared to normal weight men (OR, 95% CI: 0.49, 0.46–0.52). We also performed two separate interaction analyses between BMI and parental education respective BMI and intelligence and found no statistical significant interaction (data not shown).

**Table 2:** **Odd ratios, with 95% confidence intervals, formarital status at age40 (between 1991 and2001) according to BMIand socio-demographic characteristics amongmen born between 1951and 1961 in Sweden**

	**Married**^**1**^	**Never married**^**1**^	**Divorced**^**1**^	**Widowed**^**1**^
**BMI category**^**2**^				
Underweight	0.84 (0.82-0.86)	1.00	0.92 (0.88-0.96)	0.72 (0.52-1.00)
Normal weight	1.00	1.00	1.00	1.00
Overweight	0.83 (0.80-0.85)	1.00	0.83 (0.79-0.87)	0.95 (0.67-1.33)
Obese	0.49 (0.46-0.52)	1.00	0.55 (0.49-0.63)	0.48 (0.18-1.30)
**Education**^**1**^				
High education	1.00	1.00	1.00	1.00
Medium education	0.87 (0.86-0.89)	1.00	1.31 (1.26-1.36)	0.89 (0.69-1.14)
Low education	0.81 (0.79-0.83)	1.00	1.30 (1.24-1.36)	1.08 (0.80-1.46)
**Socioeconomic position**^**3**^				
White-collar	1.00	1.00	1.00	1.00
Farmers	0.81 (0.77-0.86)	1.00	0.54 (0.47-0.63)	0.88 (0.45-1.72)
Blue-collar	0.74 (0.73-0.75)	1.00	0.90 (0.87-0.93)	0.75 (0.60-0.96)
Other	0.47 (0.46-0.48)	1.00	0.84 (0.81-0.88)	0.57 (0.41-0.78)
**Intellectual performance**^**2**^				
Lower	0.74 (0.72-0.75)	1.00	0.98 (0.94-1.02)	0.95 (0.73-1.25)
Medium	0.92 (0.91-0.94)	1.00	1.03 (0.99-1.07)	1.06 (0.82-1.35)
Higher	1.00	1.00	1.00	1.00
**Country of birth**^**3**^				
Sweden	1.00	1.00	1.00	1.00
Other	0.93 (0.88-0.98)	1.00	1.23 (1.12-1.35)	0.42 (0.13-1.30)
**Childhood socioeconomic position**^**3**^				
White-collar	1.00	1.00	1.00	1.00
Farmers	0.99 (0.96-1.02)	1.00	0.64 (0.60-0.68)	1.17 (0.81-1.68)
Blue-collar	0.97 (0.95-0.99)	1.00	0.98 (0.95-1.01)	1.03 (0.82-1.30)
Other	0.85 (0.81-0.88)	1.00	1.10 (1.03-1.17)	1.01 (0.61-1.66)
**Parental education**^**3**^				
High education	1.00	1.00	1.00	1.00
Medium education	0.99 (0.96-1.01)	1.00	1.10 (1.04-1.17)	1.00 (0.68-1.48)
Low education	0.98 (0.96-1.01)	1.00	1.02 (0.96-1.08)	0.90 (0.60-1.35)
**Residential area**^**2**^				
Urban	1.00	1.00	1.00	1.00
Semi-urban	0.98 (0.97-1.00)	1.00	0.85 (0.83-0.88)	1.06 (0.86-1.30)
Rural	0.93 (0.91-0.95)	1.00	0.76 (0.73-0.80)	0.93 (0.70-1.23)
**Psychiatric disorder before and/orat military conscription examination**^**2,4**^				
Yes	0.65 (0.63-0.66)	1.00	1.04 (1.00-1.08)	0.65 (0.46-0.90)
No	1.00	1.00	1.00	1.00
**Muscle strength at militaryconscription examination**^**2**^				
Low quintile	0.65 (0.63-0.66)	1.00	0.66 (0.63-0.70)	0.75 (0.55-1.03)
Low to mediate quintile	0.79 (0.78-0.81)	1.00	0.77 (0.74-0.80)	0.77 (0.58-1.02)
Mediate quintile	0.88 (0.86-0.90)	1.00	0.88 (0.84-0.92)	0.79 (0.59-1.07)
Mediate to high quintile	0.93 (0.91-0.95)	1.00	0.94 (0.90-0.98)	0.72 (0.53-0.98)
High quintile	1.00	1.00	1.00	1.00

Never married is the outcome reference.

^1^=LOUISE, ^2^=Military Service Conscription Register, ^3^=Population and Housing Censuses, ^4^=Hospital Discharge Register.

Fully adjusted model (n=403,923).

Many other factors markedly influenced the likelihood of being married. Having a lower education decreased the likelihood of being married (OR: 0.81, 0.79–0.83) and increased the likelihood of being divorced (OR: 1.30, 1.24–1.36), in relation to those with a higher level of education. Men born outside Sweden were slightly less likely (OR: 0.93, 0.88–0.98) to be married and more likely (OR: 1.23, 1.12–1.35) to be divorced compared to men born in Sweden. The likelihood of being married was also markedly decreased among those men who had no labour market position, the group "other" (OR: 0.47, 0.46–0.48), compared to white-collar workers. In addition, blue-collar workers were less likely to be married (OR: 0.74, 0.73–0.75) compared with white-collar workers. Intellectual performance was also a predictor of the likelihood of being married. Men with lower intellectual performance were less likely (OR: 0.74, 0.72–0.75) to be married than men with a higher intellectual performance. There was also a markedly lower likelihood of being married among men with a psychiatric disorder (OR 0.65, 0.63–0.66).

## Discussion

We conducted this study on a very large dataset of Swedish men born from 1951 to 1961, with information on BMI from military conscription examination at age 18, socioeconomic position in childhood and adulthood and marital status at age 40 years. Our results showed that men categorized as obese at age 18 were half as likely to be married at 40 years of age (on average 22 years after their military conscription examination) compared with men of normal weight. Significant associations between obesity and marital status among men in a longitudinal setting are novel and important findings. A few previous studies have reported a lower likelihood of marriage among obese women [4,17] and our findings confirm that this is also the case for obese men.

The likelihood of marriage was decreased not only among obese men, but also among underweight men, which is in line with a recent nationwide population-based study on Swedish 10-year-old children that found stereotypical attitudes and prejudice against obese peers but also against those who were underweight [18]. A higher likelihood of being married at age 40 was found among men with higher intellectual performance, higher education, white-collar workers, men with higher muscular strength at conscription examination and men with no psychiatric diagnoses at conscription examination. This is in accordance with earlier studies looking at possible selection mechanisms whereby healthier people seek healthier counterparts [5,9]. The decreased risk for getting married among men with obesity could also be explained by nutritional and genetic factors early in life, but it is unlikely that, for example, certain genes are involved in whether people get married or not.

This study has several strengths. The main strengths are that it was nationwide, population-based, longitudinal with a follow-up time of at least 20 years, and had a high rate of participation (>90%). Furthermore, the data on height, weight, socioeconomic position and education (both in childhood and as adults), and marital status were collected from population-based registers and thus were not self-reported. BMI was measured at age 18–20 years, before adult marital status had been firmly established, avoiding the possibility of reverse causality [19,20]. Men who were married before their military conscription examination were excluded to further support this argument. However, the early measurement of BMI can also be considered a limitation of the study. The prevalence of obesity was most probably higher at age 30–40 years, resulting in some degree of misclassification bias. However, this type of potential bias should have skewed the estimates toward the null hypothesis. Further, the aim of the study was to contribute to knowledge of the long-term effects of obesity, and thus the early measurement of BMI can be considered an advantage. It is, however, a weakness that our study was limited to Swedish men, making it impossible to make any inferences about women. In Sweden, military enlistment examination was only compulsory for men, and thus similar data on women are not available. It is also a limitation that we were unable to adjust for constructs such as self-esteem.

It is also likely that a rather large number of those classified as never married actually live with a partner, but have never formally married. However, since the social norm in Sweden is to live within a marriage, those that never marry can be considered to have a social disadvantage compared to their married counterparts. A further limitation is that no information was available for marital status before the year 1990. We chose marital status at age 40 years because at that age marital status is assumed to be established.

There are several potential explanations for our results. At the individual level, the mechanisms of assortative mating where like chooses like, e.g. with respect to height, education and obesity status, are well known [21]. Assortative mating would result in obese men marrying obese women more often than they would marry normal weight women. This phenomenon could explain our results if obesity was much more common among men than among women in Sweden. The differences in prevalence of obesity among men and women in Sweden are, however, not large enough to explain the results [22]. Further, obese individuals might perceive themselves, and other obese individuals, as being less attractive, or less valuable than normal-weight individuals [23]. Obese men might choose not to marry, rather than to marry a woman of their own size. Another explanation at the individual level might be low self-esteem, which is often seen among obese people [24]. Low self-esteem might lead to less confidence when dating and may be a substantial impediment to marriage. Unfortunately, no information on self-esteem has been collected in this study so we are unable to control for that in our analyses.

In a study conducted in the USA the author found that obese women were less likely to be engaged in a union, marriage or cohabiting [25]. Men with obesity on the other hand were less likely to cohabit with women but not less likely to be married, so the absence of an adverse impact of obesity for men suggests that marriage provides extra utility for women. In our study, obese men were less likely to be married at age 40 years. One explanation why Swedish obese men are penalted on the marriage market, while less evidence has been found in the U.S., might be that Swedish men are more exposed to social stigma than American men. As previously reported; Swedish men who were obese in late adolescence were less likely to receive higher education and had an increased risk of being granted a disability pension later in life compared to their normal weight counterparts [10,11]. Swedish obese men have also been shown to be more downwardly than upwardly socially mobile in the social hierarchy compared to their normal weight counterparts [26]. Stigmatization of obese people, which is a widespread phenomenon in many societies, including Sweden [18], is a serious threat against the social and economic status of obese people.

## Conclusion

The probability of being married at age 40 was lower within groups of lower status, i.e. men with obesity, lower intellectual performance, lower education, blue-collar workers, no occupational title, and men with psychiatric disorders. Our results add to an increasing body of evidence indicating that obese people’s prospects in life are not as good as those of normal weight people. Developing interventions that influence negative attitudes in society towards obese individuals thus remains a challenge. However, more knowledge is needed about the complex pathways, some of which are illustrated above, and about the many factors other than obesity, that influence the possibilities and prospects in life for obese people.

## Competing interests

The authors declare that they have no competing interests.

## Authors' contributions

MK participated in the design of the study, performed the statistical analysis and drafted the manuscript. NK conceived of the study, participated in the design and drafted the manuscript. All authors read and approved the final manuscript.

## Pre-publication history

The pre-publication history for this paper can be accessed here:

http://www.biomedcentral.com/1471-2458/12/833/prepub
